# The Role of Corticotropin-Releasing Factor (CRF) and CRF-Related Peptides in the Social Behavior of Rodents

**DOI:** 10.3390/biomedicines11082217

**Published:** 2023-08-07

**Authors:** Zsolt Bagosi, Kíra Megyesi, Jázmin Ayman, Hanna Rudersdorf, Maieda Khan Ayaz, Krisztina Csabafi

**Affiliations:** 1 Department of Pathophysiology, Albert Szent-Györgyi School of Medicine, University of Szeged, 6720 Szeged, Hungary; hanna.rudersdorf@icloud.com (H.R.); maieda.kh@hotmail.com (M.K.A.); csabafi.krisztina@med.u-szeged.hu (K.C.); 2 Interdisciplinary Center for Excellence, Clinical Research Competence Center, Albert Szent-Györgyi School of Medicine, University of Szeged, 6720 Szeged, Hungary; megyesikira@gmail.com; 3 Department of Obstetrics and Gynecology, Albert Szent-Györgyi Albert School of Medicine, University of Szeged, 6720 Szeged, Hungary; aymanjazmin@gmail.com

**Keywords:** CRF, urocortin, CRF receptor, social interaction

## Abstract

Since the corticotropin-releasing factor (CRF) was isolated from an ovine brain, a growing family of CRF-related peptides has been discovered. Today, the mammalian CRF system consists of four ligands (CRF, urocortin 1 (Ucn1), urocortin 2 (Ucn2), and urocortin 3 (Ucn3)); two receptors (CRF receptor type 1 (CRF1) and CRF receptor type 2 (CRF2)); and a CRF-binding protein (CRF-BP). Besides the regulation of the neuroendocrine, autonomic, and behavioral responses to stress, CRF and CRF-related peptides are also involved in different aspects of social behavior. In the present study, we review the experiments that investigated the role of CRF and the urocortins involved in the social behavior of rats, mice, and voles, with a special focus on sociability and preference for social novelty, as well as the ability for social recognition, discrimination, and memory. In general, these experiments demonstrate that CRF, Ucn1, Ucn2, and Ucn3 play important, but distinct roles in the social behavior of rodents, and that they are mediated by CRF1 and/or CRF2. In addition, we suggest the possible brain regions and pathways that express CRF and CRF-related peptides and that might be involved in social interactions. Furthermore, we also emphasize the differences between the species, strains, and sexes that make translation of these roles from rodents to humans difficult.

## 1. Introduction

Since the corticotropin-releasing factor (CRF) was isolated from an ovine brain, a growing family of CRF-related peptides has been discovered [1]. Today, the mammalian CRF system consists of four ligands (CRF) [2], urocortin 1 (Ucn1) [3], urocortin 2 (Ucn2) [4], and urocortin 3 (Ucn3) [5]; two receptors (CRF receptor type 1 (CRF1) [6] and CRF receptor type 2 (CRF2) [7]); and a CRF-binding protein (CRF-BP) [8]. These CRF-related peptides are implicated in the regulation of the neuroendocrine, autonomic, and behavioral responses to stress [9,10,11].

The neuroendocrine stress response is represented by the activation of the hypothalamic–pituitary–adrenal (HPA) axis, and it is initiated by hypothalamic CRF, which—along with the synergistic arginine vasopressin (AVP)—is synthesized and released from the paraventricular nucleus (PVN) and stimulates the release of corticotropin (also known as adrenocorticotropic hormone (ACTH)) in the anterior pituitary (APit) [12,13,14]. ACTH, in turn, stimulates the synthesis and release of glucocorticoids from the adrenal cortex, which consists mainly of cortisol in humans and corticosterone in rodents [12,13,14]. The autonomic stress response is represented by the activation of the sympathetic nervous system (SNS). This system is mediated by extrahypothalamic CRF [12,13,14] and catecholamines—such as the noradrenaline released from the locus coeruleus (LC) and the adrenaline released from the adrenal medulla—which result in the so-called “fight or flight or freeze” reaction [12,13,14]. The behavioral stress response includes stimulation of locomotion in a familiar environment, the suppression of locomotion in an unfamiliar environment, reduction in food and water intake, and the inhibition of sexual and social behavior, which are promoted by both hypothalamic and extrahypothalamic CRF [12,13,14].

**Figure 1:**
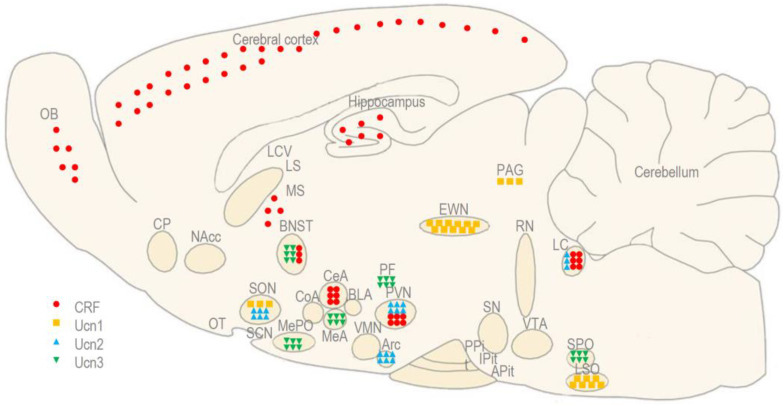
The anatomical distribution of CRF and the urocortins (Ucn1, Ucn2, and Ucn3) in the rodent brain. Adapted with permission from Ref. [21].

CRF is a 41-amino acid peptide that was isolated from an ovine brain in 1981, but has also been detected in rodents, primates, and humans [2]. Human CRF presents a 54% homology with the urotensin from fish urophysis, and a 48% homology with the sauvagine from frog skin [1]. In mammals, CRF is synthesized predominantly in the paraventricular nucleus (PVN) of the hypothalamus and in the central nucleus of the amygdala (CeA), but it is also expressed abundantly in the cerebral cortex, olfactory bulb (OB), medial septum (MS), the bed nucleus of stria terminalis (BNST), and LC [2,15,16] (Figure 1). CRF acts preferentially through CRF1, binding with a 15-fold higher affinity to CRF1 than to CRF2 [9]. The administration of CRF induces the activation of the HPA axis [15], stimulation of locomotion (at least in a familiar environment), and a reduction in food and water intake [10,11,16].

Ucn1 is a 40-amino acid neuropeptide that was isolated from the rat brain in 1995 [3]. Ucn1 presents a 63% homology with fish urotensin and a 45% homology with human CRF. In addition, even its name was derived from these two peptides (urotensin + corticotropin-releasing factor = urocortin) [1]. In mammals, Ucn1 is expressed predominantly in the lateral superior olivary nucleus (LSO) and the urocortin-synthesizing part of the Edinger–Westphal nucleus (EWN), known as the centrally projecting EWN (EWNcp) [3,17,18,19,20,21,22,23,24] (Figure 1). Unlike the classical pre-ganglionic EWN (EWNpg), which participates in oculomotor function, the EWNcp modulates the neuroendocrine and behavioral responses to stress and alcohol intake [25,26,27,28,29]. Ucn1 acts equipotently through both CRF receptors, it binds with a 7-fold higher affinity to CRF1 and a 40-fold higher affinity to CRF2 than to CRF itself [16]. The administration of Ucn1 produces a similar stimulation of the pituitary ACTH release [15], a less potent stimulation of locomotion, and a more potent reduction in food and water intake when compared to CRF [10,11,16,30,31,32,33,34]. 

Ucn2 is a 38-amino acid neuropeptide that was first identified in a mouse brain in 2001 [4]. In humans, it is also known as a stresscopin-related peptide, presenting a 34% homology with human CRF [1]. Ucn2 is expressed in the PVN and the LC, suggesting its role in the regulation of the HPA axis and SNS, but also the supraoptic nucleus (SON) and arcuate nucleus (Arc) of the hypothalamus, which modulate food and water intake [35,36] (Figure 1). The actions of Ucn2 are mediated exclusively by CRF2 [9], and it has a 100-fold higher affinity for CRF2 than for CRF1 [9]. The administration of Ucn2 induces mild locomotor suppressive and delayed anxiolytic-like actions, as well as a reduction in food and water intake [37,38].

Ucn3 is another 38-amino acid neuropeptide that was identified in a mouse brain in 2011 [5]. In humans, it is also known as stresscopin, presenting a 36% homology with human CRF [1]. Ucn3 is expressed in the perifornical area of the hypothalamus (PF), which is adjacent to the PVN and in many other brain regions, such as the BNST, lateral septum (LS), medial amygdala (MeA), and superior paraolivary nucleus (SPO), which are reciprocally connected with the PVN, suggesting its role in coping with stress [36,39] (Figure 1). The actions of Ucn2 are mediated exclusively by CRF2 [9], and it has a 1000-fold higher affinity for CRF2 than for CRF1 [9]. The administration of Ucn3 produces mild locomotor suppressive and anxiolytic-like actions, as well as reduces food intake and gastric emptying, even more potently than Ucn2 [40,41].

CRF1 and CRF2 belong to the class B subtype of G protein-coupled receptors, and—like such—they are composed of an amino-terminal extracellular region, seven transmembrane segments connected by alternating intracellular and extracellular loops, and a carboxyl-terminal intracellular tail [42].

**Figure 2:**
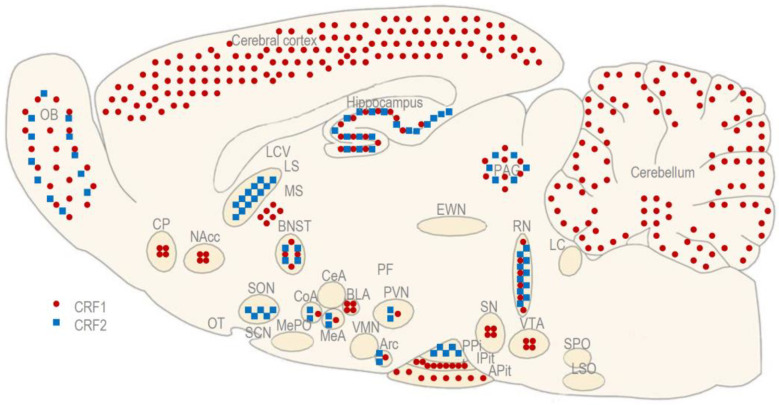
The anatomical distribution of CRF receptors (CRF1 and CRF2) in a rodent brain. Adapted with permission from Ref. [21].

CRF1 is a protein that consists of 415 amino acids, and it has two functional isoforms, α and β. Both isoforms have also been described in rodents, primates, and humans [6,10]. CRF1 is more abundant in the central nervous system (CNS) than in the periphery. In the brain, it is distributed widely in the cerebral cortex, cerebellum, and APit [43] (Figure 2).

CRF2, consisting of 437 amino acids, has three functional isoforms, α, β, and γ, but only the first two isoforms have been described in rodents, primates, and humans [7,10]; the third one has only been reported in humans [37]. CRF2 is more abundant in the periphery than in the CNS. Centrally, it is limited to subcortical regions, including the MeA, BNST, hippocampus, LS, and posterior pituitary (PPit) [36] (Figure 2).

CRF-BP is a 322-amino acid protein found in rodents, primates, and humans [8,44,45,46,47]. In rodents and primates, it has been shown in the cerebral cortex, hypothalamus, and APit [48]; in humans, it has also been found in the liver and in circulation [8,44,45,46,47]. The role of CRF-BP is not clear. It may act as a binding protein for CRF and Ucn1, inhibiting their effects during pregnancy, or as an escort protein for CRF2, potentiating the effects of CRF and Ucn1 [49,50].

The activation of CRF1 by the intracerebroventricular (ICV) injection of CRF and Ucn1 evokes the activation of the HPA axis, as well as anxiety-like and depression-like behavior [2,17,18], whereas activation of CRF2 by the ICV administration of Ucn2 and Ucn3 has anxiolytic and antidepressant effects in rats [19,20,21,22]. Therefore, originally, it was presumed that CRF1 and CRF2 exert dualistic effects in the CNS [2,3,4,5]. During stress, CRF is released from the PVN, and it stimulates the release of ACTH from the APit via CRF1 [12,13]. Subsequently, pituitary ACTH stimulates the synthesis and release of glucocorticoids, which is represented mainly by corticosterone in rodents and cortisol in humans [12,13,14]. The elevation of the plasma ACTH and glucocorticoid levels not only reflects the activation of the HPA axis, but also exerts a negative feedback effect on the release of hypothalamic CRF, inhibiting the HPA axis [12,13]. CRF-BP may also inhibit CRF and Ucn1 during physiological states, such as pregnancy or excessive stress. Excessive stress may produce a pathological stimulation of the CRF/CRF1 system in the cerebral cortex and amygdala, which overrules the urocortin/CRF2 system in the LS and hippocampus, leading to stress-related diseases, such as anxiety and depression [51,52]. However, the ICV administration of Ucn1, Ucn2, and Ucn3 in mice, and the global knock-out of urocortins and their receptors led to different results regarding the activation of the HPA-axis, as well as for anxiety- and depression-like behavior [53]. Moreover, based on modern experimental techniques—such as CRF over-expression and the site-specific knock-out of CRF1 and CRF2—it was recently proposed that the role of CRF1 and CRF2 in stress, anxiety, and depression is not a matter of simple dualism, but that it depends on the brain regions and neuronal populations being activated [54,55].

Besides the regulation of the neuroendocrine, autonomic, and behavioral responses to stress, CRF and CRF-related peptides have been involved in different aspects of social behavior [56,57]. Previous studies have already reviewed the impacts of social stress, such as social isolation and social defeat on the CRF system [57], as well as the effects of the CRF system on social behavior, including pair bonding, affiliative, and aggressive behavior, and also maternal defense and parental care [56]. The experiments summarized in these studies were performed on a variety of species, ranging from fishes, frogs, and birds to mammals [56,57]. In the present study, we review the experiments that investigated the role of CRF and urocortins in the social interaction of rats, mice, and voles, with a special focus on sociability, preference for social novelty, as well as the ability for social recognition, discrimination, and memory. Rodents regularly interact with each other socially in order to survive individually and to perpetuate the species [58]. Such social interactions require a motivation to explore the conspecifics [59,60] of the same or opposite sex (termed sociability and preference for social novelty), as well as the ability to recognize, discriminate, and remember specific individuals (termed social recognition, discrimination, and memory), which can be investigated in a one-chamber social interaction box, a three-chamber social interaction box, or in the home cage of the tested animal [58].

## 2. Experiments in Rats

To examine the role of CRF and urocortins in the social behavior of rats, male or female rats of different strains were injected with ICV directly into the BLA or BNST with CRF or Ucn1, and they were then investigated in a social interaction test for anxiety-like and social behavior [61,62,63,64,65,66,67,68,69] (Table 1). In this test, a one-chamber social interaction box (L × W × H: 65 × 65 × 47 cm) was used [70]. First, the tested male animal was familiarized with the social interaction box for 10 min [70]. Then, the animal was exposed to a male or female conspecific, and the duration of active or passive social contacts was measured for 10 min [70]. Active social contact was considered when the animals were sniffing, nipping, grooming, following, mounting, kicking, boxing, wrestling, crawling under, or jumping over each other [70]. Passive social contact was considered when the animals were sitting or lying with their bodies in contact [70]. Social behavior was inversely associated with anxiety-like behavior; therefore, a decrease in active or passive social interactions may indicate anxiety in the animal [70].

In one of these experiments, CRF was injected ICV in male Lister Hooded rats. CRF significantly decreased the time of active, but not passive social interaction [70]. In the next experiments, CRF or Ucn1 were injected into the BLA of male Wistar rats [62,63]. Both non-selective CRF1 agonists decreased the time of active social interaction, but Ucn1 was even more potent than CRF in doing so [62,63]. When Ucn1 was injected directly into the BLA of male Wistar rats, it induced a so-called “priming” to the panicogenic effect of intravenously (IV) injected sodium lactate, which included behavioral and cardiovascular responses [63,66]. The priming effects of Ucn1 were reversed by the non-selective CRF receptor antagonist astressin, which was co-administered into the BLA [63]. The anxiogenic actions of stress and Ucn1 on social behavior were reversed by an intraperitoneal (IP) injection of selective CRF1 antagonists, such as NBI3b1996 and antalarmin [64,67]. When Ucn1 was injected directly into the BNST of male Wistar rats, it decreased the time of active social interaction, but it did not produce cardiovascular, but only behavioral “priming” to the panicogenic effect of sodium lactate IV [66]. The behavioral effect of Ucn1 was blocked by the astressin that was co-administered into the BNST [66]. 

To determine the specific role of CRF1 and CRF2 in the social behavior of rats, male Wistar rats were exposed to an ICV injection of stressin1-A, a selective CRF1 agonist, and Ucn3, a selective CRF2 agonist, followed by several behavioral tests, including a social interaction test [68]. Stressin1-A induced anxiety-like behavior, which was reflected in social interactions, while Ucn3 did not influence social interactions [68]. When female CD rats were injected ICV with D-Phe CRF (12–41) (a competitive CRF antagonist), and r/h CRF (6–33) (a CRF-BP inhibitor)—and were then examined in a social recognition and discrimination test—both compounds dose-dependently decreased and increased the time of exploration for the juvenile during second exposure [69]. However, neither the competitive CRF antagonist, nor the CRF-BP inhibitor considerably influenced the time of exploration for the juvenile during first exposure [69].

These experiments demonstrate that CRF and Ucn1 induce anxiogenic and panicogenic behavior, as well as emphasize the role of BLA and BNST in this behavior [30,62,66]. Altogether, these experiments indicate that CRF1 and CRF2 play contrasting, but not opposite roles, in social behavior, and that CRF-BP has a similar role in social recognition and discrimination to that of CRF2 [68].

## 3. Experiments in Mice

To examine the role of CRF and CRF-related peptides in the social behavior of mice, male or female mice of different strains were injected ICV with CRF, Ucn1, Ucn2, and Ucn3, or they were directly administered into the MeA with Ucn3. The mice were then tested for their sociability and preference for social novelty (Table 2).

In the sociability and preference for social novelty test, a three-chamber social interaction box (L × W × H: 60 × 20 × 25 cm) was used [74]. First, the tested male animal was familiarized with the middle chamber for 5 min; then, the male was allowed to explore the adjacent chambers for another 5 min [71,72]. In this paradigm, two types of tests can be performed [71,72]. The first test was used to investigate the sociability of animals; in this test, the male can choose between a stranger male conspecific set in a cage and an empty cage [71,72]. The second test was used to investigate the preference for the social novelty of animals; in this test, the male can choose between a stranger male (or female) and a familiar male (or female) conspecific [71,72]. The familiar animal can be represented by a stranger male, which the tested male was familiarized with in the first test, or a familiar female, which the tested male was previously held together with for 24 h [71,72]. In both tests, the number of entries into each chamber and the duration of interaction with stranger animals, familiar animals, or the empty cage were measured [71,72]. The tested animal was considered to be in the chamber once its head and four paws had entered into the chamber, and interaction was considered when the body of the animal was in an area of 3 cm around the cage [71,72]. Social anxiety disorder, autism spectrum disorder, and schizophrenia are associated with reduced social interactions, including a reduction in sociability and a preference for social novelty [71,72].

In the first study, male Carworth Farms Lane-Petter (CFLP) mice were treated ICV with CRF, Ucn1, Ucn2, or Ucn3 alone or in combination with the selective CRF1 antagonist antalarmin or the selective CRF2 antagonist astressin2B; they were then tested for their sociability [71]. CRF significantly decreased the number of entries to and the time of interaction with the stranger male and the empty cage; furthermore, the decreasing effects were blocked by antalarmin, but not astressin2B [71]. In contrast, Ucn1 significantly increased the number of entries to the empty cage, but did not influence the time spent with the stranger male; moreover, the increasing effect was blocked by astressin2B, but not antalarmin [71]. Ucn2 and Ucn3 significantly decreased the time spent with the stranger male, but did not considerably affect the number of entries to the male [71].

In the second study, male CFLP mice were treated ICV with the same CRF agonists alone or in combination with selective CRF antagonists, and they were then investigated for their preference for social novelty [72]. CRF significantly decreased the number of entries to and the time spent with the stranger female, but not the familiar female, and the decreasing effect was blocked by antalarmin, but not astressin2B [72]. Also, Ucn1 decreased the number of entries to, but did not influence the time spent with the stranger female or the social interaction with the familiar female; in addition, the decreasing effect was blocked by the selective CRF1, but not CRF2 antagonist [72]. Ucn2 and Ucn3 did not considerably affect either the number of entries to or the time spent with the females [72].

In the third study, male C57BL/6 mice were injected with Ucn3 directly into the MeA and were then examined in a modified version of the preference for social novelty test [73]. In this test, the social interaction box was composed of three chambers: the first chamber was a remote chamber (non-social), the second contained a nest-mate sibling (the familiar male), and the third a novel conspecific (the unfamiliar male) [73]. After familiarizing with the middle chamber for 30 min, the tested male was allowed to explore the adjacent chambers for 20 min [73]. Mice injected with Ucn3 into the MeA spent more time near the unfamiliar male, less time near the familiar male, and a similar time in the remote, non-social chamber compared to the control mice. However, these effects were reversed by the selective CRF2 antagonist astressin2B [73].

These studies demonstrated that CRF and Ucn1 exert different effects on sociability, which are mediated by CRF1 and CRF2, respectively, as well as a similar effect on preference for social novelty, which is promoted by CRF1 [71,72]. These studies also indicate that Ucn2 and Ucn3 decrease sociability and increase preference for social novelty in regard to male–male interaction, but not male–female interaction; in addition, they emphasize the role of MeA in this behavior [71,72,73]. Moreover, these experiments suggest that the role of CRF2 in social interactions is site-specific and sex-dependent [71,72,73].

To determine more specifically the role of CRF1, CRF2, and CRF-BP in the social behavior of mice, male and female knock-out mice were generated from C57BL/6 mice, and were then tested for their sociability and preference for social novelty, as well as their social recognition and discrimination abilities. They were then exposed to a series of other tests based on a resident–intruder paradigm [73,75,76,77,78,79,80,81] (Table 3 continuation).

In the social recognition and discrimination test, the tested male animal was kept in its home cage (L × W × H: 39 × 24 × 16 cm) [83]. In this paradigm, two types of tests can be performed. In the first test, the ability for social recognition was examine, whereby a stranger juvenile (or female) was introduced into the home cage of the tested male for 4 min (the first exposure) [83]. Then, the stranger juvenile (or female) was removed and held individually in a cage, and after 30, 60, or 120 min, it was introduced again to the tested male for 4 min (the second exposure). In the second test, the ability for social discrimination and memory was examined. During the second exposure, two juveniles (or females) were introduced simultaneously to the tested male, one was introduced during the first exposure and other one was a stranger [83]. In both tests, the duration of olfactory investigation toward the social stimulus was measured during both exposures [83]. In the first exposure, an increased time of investigation toward the stranger reflected the ability for social recognition [83]. In the second exposure, an increased time of investigation toward the stranger reflected the ability for social discrimination [83]. A decreased time of investigation toward the social stimulus during the second exposure, compared to the first exposure, represented an intact social memory [83]. Social anxiety disorder, autism spectrum disorder, and schizophrenia can also be associated with defects in social recognition, discrimination, and memory [83].

Male, but not female, Ucn2 knock-out mice exhibited significantly more passive social interactions and less aggression compared to wild-type mice [81]. Ucn3 knock-out and CRF2 knock-out mice did not differ in terms of active or passive social interactions, but—unlike Ucn2-knock-out mice—both male and female Ucn3 knock-out mice showed enhanced social discrimination abilities compared to wild-type mice [80]. Global and MeA-specific Ucn3 knock-out and CRF2 knock-out mice displayed a decreased preference for social novelty [73]. In addition, the chemogenetic inhibition of MeA Ucn3 neurons induced pro-social behavior in mice without affecting their hierarchical organization [73]. In contrast, the optogenetic activation of the Ucn3 or pharmacological stimulation of CRF2 in the MeA led to a 7-fold increase in preference for social novelty [73]. In a more complex social setting, mice with high levels of Ucn3 in the brain actively sought out contact with strangers, even ignoring their own group [73], whereas Ucn2-deficient and CRF2-deficient mice chose to socialize mainly within their own group, i.e., avoiding contact with strangers [73].

Recently, male and female mice that were deficient in CRF2 were generated and treated ICV with the selective CRF1 antagonist antalarmin, and they were then investigated for their sociability [82]. CRF2 receptor deficiency decreased social interaction with the stranger conspecifics in female mice, but increased it in male mice [82]. Moreover, treatment with the selective CRF1 antagonist antalarmin consistently induced sociability in non-social mice, but disrupted it in social mice, and this occurred independently of CRF2 deficiency [82].

Male and female mice that were deficient in CRF1, CRF2, and CRF-BP were also investigated in a resident–intruder paradigm, in which the tested animal was kept in their home cage, and a new animal (a juvenile, a male, or a female) was introduced. Then, the number, duration, and latency time of the attacks between the resident and intruder were scored [75,76,77,78,79]. CRF1 knock-out mice exhibited significant deficits in nurturing behavior, and the only non-significant deficits were in maternal aggression. There was no alteration in inter-male aggression when compared to wild-type mice [75]. CRF2 knock-out mice expressed significant deficits in maternal aggression and no alteration in inter-male aggression when compared to wild-type mice [76]. Similarly, CRF-BP knock-out mice showed significant deficits in maternal aggression, but no difference in inter-male aggression, and this was probably mediated by the LS [78].

These studies demonstrate that Ucn2 decreases sociability and increases aggression in males but not females, and that Ucn3 induces preference for social novelty but reduces the ability for social discrimination in both males and females; as such, this underlines the role of MeA in this behavior [73,80,81]. These studies also indicate that the role of CRF1 is to modulate nurturing behavior, and that the role of CRF2 is to increase maternal aggression. Furthermore, CRF-BP has similar role in maternal behavior to that of CRF2, and it is probably mediated by the LS [75,76,77,78,79]. However, these experiments also suggest that the role of CRF2 in social interactions is site-specific and sex-dependent [73,80,81,82].

## 4. Experiments in Voles

Finally, in order to determine the role of CRF and CRF-receptors in the social behavior of voles, male voles of monogamous (e.g., prairie and pine) and non-monogamous (e.g., meadow and montane) species were injected ICV or directly into the core of NAcc (cNAcc) or caudate-putamen (CP) with CRF and were then investigated in a three-chamber social interaction box for their preference for a partner and a stranger female conspecific [84,85] (Table 4).

In these experiments, the tested male and the partner female were held together in their home cage for 3 h [84,85]. During the cohabitation period, the number and duration of social contacts between the male and female voles, as well as the number of threats, attacks, or fights were scored [84,85]. Then, the tested male was placed in the middle chamber of the social interaction box and allowed to choose between a stranger and a partner female for 3 h [84,85]. During the social interaction period, the number and duration of physical contacts with social stimuli, including nuzzling, grooming, and sitting motionless flank-to-flank, but also acts of social aggression were scored [84,85].

In the first study, the ICV injection of CRF into the lateral cerebral ventricle (LCV) of monogamous male prairie voles did not remarkably influence the number of social contacts with the partner female during the initial cohabitation period, but significantly increased the time of physical contacts with a partner female during the social interaction period when compared to the same with a stranger [84]. The ICV coinjection of α-helical CRF9-41, a non-selective CRF antagonist, reduced the socializing effect of CRF [84]. 

In the second study, CRF was injected directly into the NAcc of monogamous male prairie voles, and this significantly increased the number of social contacts with a partner female during the cohabitation period [85]. In contrast, when CRF was injected into the CP of male prairie voles or into the NAcc of non-monogamous meadow voles, it did not affect the number of physical contacts with the partner female [85]. Coinjection of CP-154,526 (a selective CRF1 antagonist) or anti-sauvagine-30 (a selective CRF2 antagonist) into the NAcc reversed the facilitating effect of CRF [85].

Overall, these studies demonstrate that CRF facilitates partner preference by activating CRF1 and/or CRF2 when injected ICV or site-specifically into monogamous species, but not in non-monogamous species. Furthermore, this emphasizes the role of the NAcc, but not CP, in this process [84,85].

## 5. Discussion

**Figure 3:**
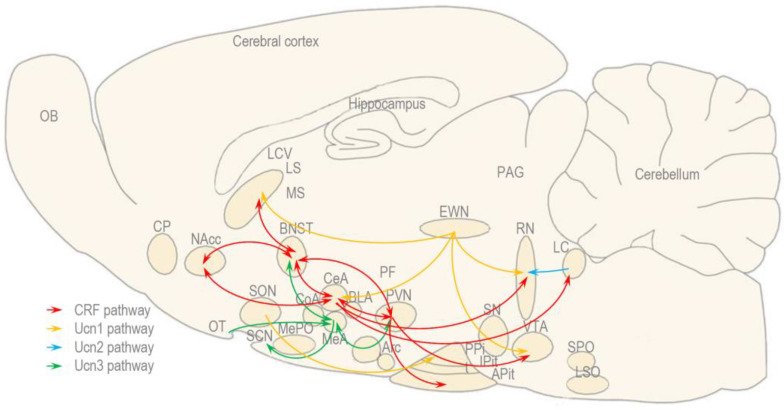
The brain regions and pathways that express the CRF and CRF-related peptides involved in the social behavior of rodents. Adapted with permission from Ref. [21].

In the present study, we reviewed the experiments investigating the role of CRF and urocortins in the social behavior of rats, mice, and voles, with a special focus on sociability and preference for social novelty, as well as the ability for social recognition, discrimination, and memory [30,61,62,63,64,65,66,67,68,69,70,71,72,73,75,76,77,78,79,80,81,82,84,85]. In addition, we suggest the possible brain regions and pathways that express CRF and CRF-related peptides that might be involved in social interactions (Figure 3).

CRF induces anxiogenic and panicogenic behavior, and this is mediated by BLA or CRF, which induce anxiogenic and panicogenic behavior that is mediated by BLA or BNST [62,67]. In particular, CRF neurons in the BNST are known to have multiple connections with brain regions, such as PVN and LS, and they are involved in both stress response and social behavior [59,60] (Figure 3). Consistently, CRF decreases the sociability and preference for social novelty, as well as facilitates partner preference, at least in monogamous species [84,85]. The anxiety-like behavior induced by CRF and Ucn1 can be mediated by both hypothalamic and extrahypothalamic CRF [51,86]. Hypothalamic CRF, synthesized and released from the PVN, stimulates the activity of the HPA axis, and this is reflected by the release of ACTH from the APit, which—in turn—stimulates the synthesis and release of the glucocorticoids from the adrenal cortex leading to locomotor suppression in a familiar environment [12,13,14]. Extrahypothalamic CRF that is released from the CeA can stimulate the noradrenaline release from the LC, and it can inhibit the serotonin release in the raphe nuclei (RN), which results in the “fight or flight or freeze” reaction that can affect social behavior [51,52]. CRF neurons from the PVN send projections toward the ventral tegmental area (VTA) that activates the mesolimbical pathway, which results in the release of dopamine in the NAcc and can facilitate partner preference [85,87]. Also, the paraventricular neurons have reciprocal connections with the CeA, BNST, and the shell of NAcc (shNAcc), which are known together as the extended amygdala circuit, and which represent an interface between the stress response and social behavior [59,60,88]. 

Ucn1 also induces anxiogenic and panicogenic behavior, but this behavior is mediated mainly by the BLA [30,62,66]. Ucn1 exerts a different effect on sociability and a similar effect on the preference for social novelty when compared to CRF, and this could be related to the EWNcp [71,72]. Ucn1 neurons in the EWNcp send projections to the LS, CeA, VTA, and RN where Ucn1 can stimulate the release of CRF, GABA, dopamine, and serotonin, thereby resulting in complex social dynamics [9,16] (Figure 3). For example, dopamine and serotonin can mediate positive, affiliative, but also negative, aggressive behavior [89,90,91,92,93,94,95,96,97]. Ucn1-synthesizing neurons can also modulate social behavior by inducing synthesis and/or the release of AVP, as well as oxytocin locally in the SON or distally in the PPit [9,16]. AVP and oxytocin have antagonistic effects on the stress response, but they both facilitate bond formation and partner preference [98,99,100,101]. 

Ucn2 plays a minor role in social interactions [71,72,80,81]. The only significant action of Ucn2 on social behavior, is that it increases the time of social interaction between males that could be related to Ucn2-expressing neurons in the LC, which project to the RN that expresses CRF2 [9,10,11] (Figure 3). As we previously mentioned, the dopamine and serotonin released from these nuclei can mediate both affiliative and aggressive behavior [89,90,91,92,93,94,95,96,97].

Ucn3 plays a major role in social behavior, but its actions seem to depend on the brain regions and neuronal populations being activated [68,71,72]. When administered ICV, Ucn3 decreases sociability, but it does not influence the preference for social novelty [71,72]. In contrast, when administered directly into the MeA, Ucn3 increases the preference for social novelty [71,72,73]. Also, the manipulation of Ucn3 and CRF2 in the MeA alters the ability for social discrimination [73,80]. The MeA that expresses both Ucn3 and CRF2 is particularly important in social behavior, considering that this nucleus of the amygdala is connected vice versa with the PVN and BNST, as well as the brain regions involved in the stress response and social behavior [59,60] (Figure 3). In addition, Ucn3 neurons in the MeA send axons to the suprachiasmatic nucleus (SCN) and receive dendrites from the OT, and these connections may also differently influence social interactions [73,88]. 

The effects of CRF and Ucn1 seem to be mediated by the CRF1 and/or CRF2 expressed in the BLA and BNST [62,66,67,84]. Consequently, the activation of CRF1 by CRF and Ucn1 decreases the sociability and the preference for social novelty [71,72]. Another role of CRF1 is to modulate nurturing behavior and to facilitate partner preference [75,76,77,78,79]. The effects of Ucn2 and Ucn3 appear to be promoted by the CRF2 expressed in brain regions, such as in the MeA and LS [71,72,73,80,81,82]. However, the role of CRF2 in social interactions is site-specific and sex-dependent. Moreover, the role of CRF2 in social interactions may also depend on the type and dose of the agonist being administered, since the activation of CRF2 by Ucn1 increases sociability, activation by Ucn2 decreases it [68,71], and activation by Ucn3—in certain circumstances—increases the preference for social novelty [72,73]. An additional role for CRF2 is to mediate maternal aggression and to facilitate partner preference together with CRF1 [76,85]. CRF-BP plays a similar role to CRF2 in social recognition and discrimination, as well as in maternal behavior [78]; therefore, it may instead act as an escort protein for CRF2 than as a binding-protein for CRF and Ucn1 [49].

Originally, it was believed that the activation of CRF1 and CRF2 exert dualistic effects in regard to the activation of the HPA axis, as well as in anxiety-like and depression-like behavior, and this could also be reflected in social behavior [2,3,4,5]. However, taken together, these experiments indicate that CRF1 and CRF2 play contrasting, but not necessarily opposite, roles in social behavior [68]. Therefore, we propose that the role of CRF1 and CRF2 in social interactions is not a matter of simple dualism, but rather that it depends on the brain regions and pathways being activated [54,55] (Figure 3). Nevertheless, the brain regions and pathways that express CRF and CRF-related peptides should not be regarded as a rigid system that correspond to a certain type of social behavior, but rather as a plastic network that can be recruited in multiple social interactions, depending on the actual social context [59,60].

Species-, strain-, and sex-related differences in the anatomical distribution of CRF and CRF-related peptides may also contribute to the contrasting results regarding the social behavior of rodents [56,57,58]. However, double-labeled immunohistochemistry studies demonstrated that the distribution of CRF and urocortins is conserved across species, including in rats and mice [10]. In all mammalian species, CRF is synthesized predominantly in the PVN of the hypothalamus and CeA [2,51,52], and Ucn1 is synthesized predominantly in the EWNcp [9,10,11]. Meanwhile, Ucn2 is expressed in the PVN, SON, and Arc of the hypothalamus, whereas LC [9,10,11] and Ucn3 are expressed in the PF, BNST, LS, MeA, and SPO, which are adjacent or reciprocally connected with the PVN [9,10,11]. Immuno- and hybridization histochemistry studies also established that the major sites of CRF1 mRNA expression are represented by the cerebral cortex, cerebellum, and APit; for CRF2 mRNA expression, they are the subcortical regions, including the MeA, BNST, hippocampus, LS, and PPit in all mammals [43]. In situ hybridization studies indicated only minor variations in the relative strength of CRF1 and CRF2 binding in several forebrain and midbrain regions between rats and mice [43]. Moreover, immunocytochemistry studies demonstrated that CRF mRNA and Ucn1 mRNA expression patterns are highly conserved throughout the brain of voles [23]. However, receptor autoradiography studies reported dramatically different CRF1 and CRF2 distribution patterns in the OB, shNAcc, LS, hippocampus, and RN between monogamous (e.g., prairie and pine) voles and non-monogamous (e.g., meadow and montane) voles; this would probably determine the markedly different social behavior of the otherwise closely related species [23,102]. Most notably, CRF1 was significantly higher in the shell of the NAcc of non-monogamous voles, and CRF2 was significantly higher in the striatum, including the NAcc of monogamous voles [102]. Also, CRF2 binding was significantly higher in the BNST of males compared to the females in all vole species, and this occurred independently of their monogamous or promiscuous lifestyle [102]. Previous studies have already demonstrated that sexual hormones can influence the expression and activity of the CRF systems that underlie the neuroendocrine, autonomic, and behavioral responses to stress. For instance, sexual hormones have been shown to stimulate the activity of the HPA axis, and this reflected by the elevation of plasma ACTH and corticosterone concentration [103]. The stress caused by immunological, physical, and/or psychological stressors elicits higher CRF and AVP mRNA expression in the PVN and the CeA of female compared to male rats [104,105]. Consequently, the sex differences in PVN CRF expression, as well as in plasma ACTH and corticosterone concentration were completely prevented by gonadectomy, that is, castration in male and ovariectomy in female rats [104]. Sex differences could be also observed in anxiety-like and depression-like behavior. For example, Ucn2-deficient female, but not male, mice displayed decreased immobility in the forced swim and tail suspension tests, as well as an increased vasopressin expression in the PVN and SON when compared to the same-sex wild-type mice [106]. However, to date, only a few studies have investigated the impact of sex on the social behavior mediated by the CRF system [82,107]. Only recently it was reported that CRF2 deficiency decreases sociability in female mice, while it increases it in male mice [82]. Furthermore, CRF2-deficient male mice are more aggressive than female mice, as shown in the resident–intruder paradigm [107]. Therefore, we reached the conclusion that the role of Ucn3 and CRF2 in social behavior is not only site-specific, but also sex-dependent.

## 6. Conclusions

In general, these experiments demonstrate that CRF, Ucn1, Ucn2, and Ucn3 play important, but distinct roles in the social behavior of rodents that are mediated by CRF1 and/or CRF2 [56,57,58]. Since they were discovered, CRF1 and CRF2 have been considered potential targets in the therapy of stress-related diseases, such as anxiety and depression [51,52]. Based on the original, dualistic hypothesis, generalized anxiety disorder and major depression disorder could be treated by the inhibition of CRF1 with selective antagonists, such as antalarmin, or via the activation of CRF2 with selective agonists, such as Ucn2 or Ucn3 [108,109]. Nevertheless, while these compounds seemed very promising in preclinical experiments, they proved ineffective in clinical trials [110,111,112,113,114,115,116,117,118,119]. This could be partly due to the fact that peptides are degraded by the gastrointestinal enzymes when administered orally, and that they fail to penetrate the blood–brain barrier when injected IV [110,111,112,113,114,115,116,117,118]. Therefore, in order to increase the efficacy of these compounds, they should be administered in form of nasal spray, and eventually administered together with enzyme inhibitors and blood–brain barrier transporters [110,111,112,113,114,115,116,117,118]. However, based on the modern, site-specific hypothesis, the role of CRF1 and CRF2 in behavioral responses is not a matter of simple dualism, but depends on the brain regions and neuronal populations being activated [54,55]. Moreover, differences between species, strains, and sexes make the translation of these roles from rodents to humans difficult [119,120].

## 7. Future Directions

Interestingly, many of the brain regions participate in the stress response and reward sensation overlap with the brain network modulating social behavior [121]. The stress response involves the PVN, BLA, hippocampus, LS, CeA, BNST, NAcc, LC, and RN [12,13,14]. The reward system includes the VTA and NAcc, as well as the BNST, CeA, and prefrontal cortex [12,13,14]. The social brain network consists of the anterior, the ventromedial (VMH), and the median preoptic nuclei (MePO) of the hypothalamus, LS, MeA, BNST, NAcc, OB, olfactory tubercle (OT), and periaqueductal gray matter (PAG) [59,60]. An interface between these systems could be represented by the CeA, BNST, and shNAcc, which are known together as the extended amygdala circuit [12,13,14]. In the future, by injecting selective CRF agonists and antagonists into the LCV or directly into these brain structures, and by performing simple or more complex social interaction tests in rodents, we might be able to understand the intimate role of CRF and CRF-related peptides in different aspects of social behavior, including social isolation and social defeat, pair bonding and reward sensation, affiliative and aggressive behavior, and in maternal defense and parental care [122,123,124,125,126,127,128,129,130,131,132,133,134]. However, most of the animal experiments reviewed in the present study focused on the role of CRF and urocortins in sociability and preference for social novelty, as well as the ability for social recognition, discrimination, and memory. By understanding the role of these neuropeptides and by targeting their receptors in social anxiety disorder, autism spectrum disorder, and schizophrenia, we might improve social interactions that are typically reduced in people suffering from such disorders [135,136,137,138,139,140].

## Figures and Tables

**Table 1:** Experiments on rats.

Animals	Materials	Methods	Results	References
Male Lister Hooded rats	CRF	ICV administration followed by social interaction test	CRF decreased the time of active social interaction.	Dunn and File, 1987 [61]
Male Wistar rats	CRF and Ucn1	Single and repeated administration into the BLA followed by social interaction test	CRF and Ucn1 decreased the active social interaction time and induced cardiovascular and behavioral “priming” to the panicogenic effect of IV sodium lactate.	Sajdyk et al., 1999 [62], Sajdyk and Gehlert, 2000 [63], Rainnie et al., 2004 [65], Gehlert et al., 2005 [64], Spiga et al., 2006 [30], Shekhar et al., 2011 [67]
Male Wistar rats	Ucn1	Single and repeated administration into the BNST followed by social interaction test	Ucn1 decreased the time of active social interaction, but did not induce cardiovascular, but only behavioral “priming” to the panicogenic effect of IV sodium lactate.	Lee et al., 2008 [66]
Male Wistar rats	Stressin_1_-A and Ucn3	ICV administration followed by several tests, including social interaction test	Stressin_1_-A, but not Ucn3, decreased the time in active social interaction.	Zhao et al., 2007 [68]
Female CD rats	CRF1, CRF2, and CRF-BP	ICV administration followed by social recognition test	D-Phe CRF (12–41) and r/h CRF(6–33) induced similar alterations in recognition abilities.	Heinrichs et al., 2003 [69]

**Table 2:** Experiments in mice.

Animals	Materials	Methods	Results	References
Male CFLP mice	CRF, Ucn1, Ucn2, and Ucn3	ICV administration followed by sociability test	CRF decreased the sociability, whereas Ucn1 increased the sociability. Ucn2 and Ucn3 did not influence the number of entries, but decreased the time of social interaction with stranger males.	Bagosi et al., 2017 [71]
Male CFLP mice	CRF, Ucn1, Ucn2, and Ucn3	ICV administration followed by preference for social novelty test	CRF and Ucn1 similarly decreased the preference for social novelty. Ucn2 and Ucn3 did not influence the number of entries and the time of social interaction with stranger females.	Bagosi et al., 2017 [72]
Male and female C57BL/6 mice	Ucn3	Single administration into the MeA followed by a preference for social novelty test	Ucn3 decreased the time of social interaction with the familiar male, and increased the time of social interaction with stranger males.	Shemesh et al., 2016 [73]

**Table 3:** Continuation: Experiments in mice.

Animals	Materials	Methods	Results	References
Male and female C57BL/6J mice	Ucn2	Generation of Ucn2, Ucn3, and CRF2 knock-out mice followed by social interaction test	Male, but not female, Ucn2 knock-out mice exhibited more passive social interactions and less aggression.	Breu et al., 2012 [81]
Male and female C57BL/6J mice	Ucn2, Ucn3 and CRF2	Generation of Ucn2, Ucn3, and CRF2 knock-out mice followed by several tests, including a social recognition and discrimination test	Both male and female Ucn3 and CRF2 knock-out mice, but not Ucn2 knock-out mice, expressed enhanced discrimination abilities.	Deussing et al., 2010 [80]
Male and female C57BL/6J mice	Ucn3 and CRF2	Generation of global Ucn3 and CRF2 knock-out mice, as well as MeA-specific Ucn3 knock-out mice, followed by several tests, including sociability and preference for social novelty tests	Global Ucn3 and CRF2 knock-out mice, as well as MeA-specific Ucn3 knock-out mice displayed decreased preference for social novelty.	Shemesh et al., 2016 [73]
Male and female C57BL/6J mice	CRF1 and CRF2	Generation of CRF2 knock-out mice and the ICV administration of the CRF1 antagonist antalarmin, followed by sociability and preference for social novelty tests	CRF2 deficiency decreased sociability in female, but increased it in male mice. CRF1 antagonism induced sociability in non-social mice, but disrupted it in social mice.	Piccin and Contarino, 2020 [82]
Male and female C57BL/6J mice	CRF1, CRF2 and CRF-BP	Generation of CRF1, CRF2, and CRF-BP knockout mice, followed by a series of tests based on the resident–intruder paradigm	CRF1 knock-out mice exhibited significant deficits in nurturing and non-significant deficits in maternal aggression. CRF2 and CRF-BP knock-out mice expressed significant deficits in maternal aggression. Inter-male aggression was unaltered in all knock-out mice.	Gammie and Stevenson, 2006 [79], Gammie et al., 2007 [75], Gammie et al., 2005 [76], Gammie et al., 2008 [78]

**Table 4:** Experiments in voles.

Animals	Materials	Methods	Results	References
Male prairie voles	CRF	ICV administration followed by preference for social novelty test	CRF increased the preference for a partner during the social interaction period in monogamous prairie voles.	DeVries et al., 2002 [84]
Male prairie and meadow voles	CRF	Administration into the NAcc and CP followed by preference for social novelty test	CRF increased the preference for partners during the cohabitation period when injected into the NAcc (but not the CP) of monogamous prairie voles (but not non-monogamous meadow voles).	Lim et al., 2007 [85]

## Data Availability

Not applicable.

## Abbreviations

Arc	arcuate nucleus of the hypothalamus
APit	anterior pituitary
BLA	basolateral amygdala
BNST	bed nucleus of stria terminalis
CeA	central amygdala
CFLP	Carworth Farms Lane-Petter
cNAcc	core of nucleus accumbens
CoA	cortical amygdala
CP	caudate-putamen
CRF	corticotropin-releasing factor
CRF1	corticotropin-releasing factor receptor type 1
CRF2	corticotropin-releasing factor receptor type 2
ICV	intracerebroventricular
IP	intraperitoneal
IV	intravenous
EWN	Edinger–Westphal nucleus
EWNcp	centrally-projecting Edinger–Westphal nucleus
EWNpg	pre-ganglionic Edinger–Westphal nucleus
LC	locus coeruleus
LCV	lateral cerebral ventricle
LS	lateral septum
LSO	lateral superior olivary nucleus
MeA	medial amygdala
MePO	median preoptic area
MS	medial septum
NAcc	nucleus accumbens
OB	olfactory bulb
OT	olfactory tubercle
PAG	periaqueductal gray matter
PF	perifornical area of the hypothalamus
PPit	posterior pituitary
PVN	paraventricular nucleus of the hypothalamus
RN	raphe nuclei
SCN	suprachasmatic nucleus
SN	substantia nigra
SON	supraoptic nucleus of the hypothalamus
SPO	superior paraolivary nucleus
shNAcc	shell of nucleus accumbens
Ucn1	urocortin 1
Ucn2	urocortin 2
Ucn3	urocortin 3
VMN	ventromedial nucleus of the hypothalamus
VTA	ventral tegmental area
